# The Contribution of Autoantibodies to Inflammatory Cardiovascular Pathology

**DOI:** 10.3389/fimmu.2018.00911

**Published:** 2018-04-27

**Authors:** Lee A. Meier, Bryce A. Binstadt

**Affiliations:** Center for Immunology, Department of Pediatrics, University of Minnesota Medical School, Minneapolis, MN, United States

**Keywords:** cardiovascular, inflammation, autoantibodies, atherosclerosis, autoimmunity

## Abstract

Chronic inflammation and resulting tissue damage underlie the vast majority of acquired cardiovascular disease (CVD), a general term encompassing a widely diverse array of conditions. Both innate and adaptive immune mechanisms contribute to chronic inflammation in CVD. Although maladies, such as atherosclerosis and cardiac fibrosis, are commonly conceptualized as disorders of inflammation, the cellular and molecular mechanisms that promote inflammation during the natural history of these diseases in human patients are not fully defined. Autoantibodies (AAbs) with specificity to self-derived epitopes accompany many forms of CVD in humans. Both adaptive/induced iAAbs (generated following cognate antigen encounter) and also autoantigen-reactive natural antibodies (produced independently of infection and in the absence of T cell help) have been demonstrated to modulate the natural history of multiple forms of CVD including atherosclerosis (atherosclerotic cardiovascular disease), dilated cardiomyopathy, and valvular heart disease. Despite the breadth of experimental evidence for the role of AAbs in CVD, there is a lack of consensus regarding their specific functions, primarily due to disparate conclusions reached, even when similar approaches and experimental models are used. In this review, we seek to summarize the current understanding of AAb function in CVD through critical assessment of the clinical and experimental evidence in this field. We additionally highlight the difficulty in translating observations made in animal models to human physiology and disease and provide a summary of unresolved questions that are critical to address in future studies.

## Introduction

Cardiovascular (CV) disease (CVD) has been the most significant cause of morbidity and mortality worldwide for over a century and will continue to be for the foreseeable future (1). CVDs are heterogeneous and include coronary heart disease (CHD), peripheral vascular disease (PVD), valvular heart disease (VHD), and stroke. The main pathological process that underlies the majority of these CVD manifestations is atherosclerosis, a chronic inflammatory response to lipid products in the walls of large and medium arteries. Atherosclerosis is the single most significant contributor to human mortality (2). It has long been hypothesized that immune dysregulation and chronic inflammation contribute to the development of CV pathology independently of traditional atherosclerotic cardiovascular disease (ASCVD) risk factors (3). Until recently, however, there was no direct clinical evidence supporting the detrimental role for inflammation in this process. Outcomes from the randomized, multicenter Canakinumab Anti-inflammatory Thrombosis Outcome Study (CANTOS), completed in late 2017, provide the strongest evidence to date in support of the pro-atherogenic role for inflammation in humans. Canakinumab, a monoclonal antibody (mAb) directed against interleukin-1β (IL-1β), significantly reduced adverse CV outcomes in patients with a history of myocardial infarction (MI) and elevated C-reactive protein (CRP) (4). While, CANTOS focused solely on the effects of IL-1β blockade in secondary CV prevention, this intervention represents only one from many potential therapeutic approaches. The CANTOS trial provides confirmation of the “inflammation hypothesis” in CVD through robust clinical analysis, a critical milestone on the path toward a more comprehensive understanding of the role of inflammation in CV pathology. Although the additional clinical trials are currently underway that target other inflammatory mediators in CVD (5, 6), many unresolved questions remain regarding the specific cellular and molecular immune mechanisms that promote chronic CV inflammation. Therefore, a significant challenge facing the field of CVD research is to define these critical immune mediators, particularly those that can be targeted therapeutically.

The specific contributions that humoral immunity (e.g., complement, antibodies (Ab), etc.) provides during the natural history of CVD remain unresolved. Multiple clinical studies have demonstrated correlative evidence in favor of a CVD-promoting role for Abs, and this topic has been reviewed extensively elsewhere (7–10). Despite this, no current therapeutic approaches are designed to improve CVD outcomes by reducing Ab production or activity. Ab with reactivity to self-epitopes [autoantibodies (AAbs)] have been observed in many forms of CVD, and have diverse epitope reactivities, binding affinities, and isotypes. Abs specific to multiple varieties of cardiac/myocardium- and blood vessel-related epitopes have been characterized in human CVD, including those demonstrating binding affinity to antigens that are cardiac-specific [e.g., cardiac troponin-I (cTnI) (11)], cardiac-associated [e.g., oxidized apolipoproteins (12)], and ones that are ubiquitously expressed [e.g., heat-shock proteins (HSPs) (13)]. Despite the breadth of evidence demonstrating correlations between serum AAb titers and CVD severity, there is no consensus on the specific roles that AAbs play in CVD progression or whether they might be appropriate targets for CVD treatment. In short, contradictory evidence exists. In addition, determining whether AAbs represent causative agents rather than passive bystanders during the natural history of CVD is a challenging task, particularly in the context of highly heterogeneous manifestations of CVD in humans.

The potential mechanisms by which AAbs may promote CVD include target opsonization and subsequent recognition and activation of immune cells bearing antibody-recognizing Fc receptors (i.e., type II hypersensitivity), leukocyte activation following immune complex deposition and complement fixation (i.e., type III hypersensitivity), and target neutralization/inhibition. The purpose of this review is to summarize the current understanding how AAbs contribute to specific forms of CV pathology including ASCVD, dilated cardiomyopathy (DCM), and VHD. In addition, we highlight the key recent experimental and clinical findings in this field. Finally, we discuss a number of the remaining unresolved questions this field faces in pursuit of future clinical translation.

## The Response to CV Damage has Genetic and Environmental Contributions

The induction of an immune response to autoantigens in the setting of human CVD is thought to occur as a result of CV insults (e.g., MI and atherosclerotic plaque necrosis). Self-antigens that are normally sequestered within the cardiac parenchyma and vascular walls are liberated and/or produced during the course of an inflammatory response and its resolution. Exposure of these immunogenic elements induces innate and adaptive immune activation. Coupled with the potent inflammatory signals that invariably accompany tissue damage, robust immunopathology of CV tissue can ensue, including AAb production. Because these self-antigens are present in virtually unlimited supply, chronic autoimmunity and tissue inflammation can result.

The primary determinants of the magnitude of the induced response to self-antigens include the characteristics of the tissue insult (e.g., infarct size and microbial burden) and the affected individual’s degree of genetic predisposition to autoimmunity (14). Experimental studies in mice have provided evidence for the contribution of genetics to the development of CV pathology. For example, the A/J mouse strain is highly susceptible to enterovirus-induced experimental myocarditis whereas C57BL/6 mice are protected (15). Juvenile male BALB/c mice develop more dramatic experimental enterovirus-induced myocarditis than females (16), and atherosclerosis occurs most readily in the C57BL/6 background whereas the CH3 and BALB/c backgrounds are protected from this disease (17). The homogeneous genetic backgrounds in inbred mouse strains amplify the genetic contribution to experimental CVD initiation and progression while minimizing the contribution of environmental factors, as opposed to the diverse forms of CVD that occur in the extensively outbred human population. For example, monozygotic human twins generally develop autoimmune disease with much less than 50% concordance, underscoring the putative role for environmental factors (18). In addition, experimental animal housing conditions generally involve isolation from environmental inputs. While this strict environmental control improves the reliability and reproducibility of animal studies, and it does not accurately represent the diverse environmental stimuli that humans encounter.

The hypothesis that CV damage is a critical predecessor of AAb generation in CVD is widely accepted and supported by experimental and clinical evidence. However, this hypothesis is complicated by the observation that cardiac AAbs can also be found in apparently healthy individuals without a personal history of CVD, and the presence of these AAbs predicts the development of CVD later in life (19). In addition, the presence of CV-reactive natural antibodies (NAbs) in the general population (elaborated upon later) further complicates the understanding of the role for AAbs in CV pathology (20).

## Atherosclerosis is Driven by Inflammation

Atherosclerosis is a chronic reaction to lipid- and cholesterol-rich lipoprotein deposits (i.e., lipid- and cholesterol-rich plaques) in the sub-endothelium of large and medium arteries, and it has been reviewed extensively (3). It is the main driver of coronary artery disease (CAD), peripheral artery disease, and stroke. Of note, “cholesterol” refers to a specific chemical entity [i.e., (3β)-cholest-5-en-3-ol] but it is often conflated, out of convenience, with either low- or high-density lipoproteins (LDL and HDL, respectively). In fact, LDL and HDL are heterogeneous particles containing variable amounts of lipids and phospholipids (PLs) packaged within a combination of protein carriers (i.e., apolipoproteins). Circulating LDL and HDL have well-substantiated direct and inverse correlations, respectively, with CVD risk; because of this, significant efforts have been put into understanding the specific cellular mechanisms that underpin these observations.

The inflammatory nature of atherosclerosis is undisputed and supported by a breadth of experimental and clinical observations. It is well known that patients with systemic inflammatory diseases develop accelerated and more aggressive forms of ASCVD than the general population (21). Despite this, the mechanisms governing ASCVD initiation and progression are incompletely understood, particularly with respect to AAb generation and function. ASCVD often begins in early adolescence and is initiated by endothelial dysfunction arising primarily from disturbed hemodynamics and lipid-induced inflammation, in addition to additional environmental factors and the individual’s genetic susceptibility. The formation of macroscopic “fatty streaks” at arterial branch points and other sites of turbulence heralds the early stages of ASCVD. Fatty streaks are primarily composed of oxidized lipoprotein particles [including oxidized low-density lipoprotein (oxLDL)], foam cells (lipid-laden macrophages), vascular smooth muscle cells (vSMCs), and lymphocytes. Over an individual’s lifetime, the streak composition and structural features evolve due to chronic superimposed inflammatory and healing responses. Late-stage disease ultimately results in formation of an atheromatous plaque (22). Atherosclerosis manifests clinically due to the effects of tissue ischemia and/or infarction caused by partial or complete plaque occlusion of arterial lumens. While most commonly associated with the myocardium, atherosclerotic ischemia and infarction can affect any of the body’s tissues (e.g., in the setting of PVD).

Efforts to understand the pathophysiology of ASCVD have largely relied on one of two mouse models of the disease based on genetic disruption of lipid clearance: apolipoprotein-E- and LDL receptor-deficient mice (*ApoE*^−/−^ and *Ldlr*^−/−^, respectively). When placed on a high-fat (“Western”) diet, these mice rapidly develop extreme hyperlipidemia, and lipid-rich plaques form shortly thereafter in a predictable distribution. Genetic manipulation of these mouse models has provided significant insight into the underlying inflammatory mechanisms that promote ASCVD, with the caveat that experimental atherosclerosis in mice exhibits substantial differences from the disease in humans (23).

## B Cells Alter the Trajectory of ASCVD

The role of B cells during ASCVD initiation and progression has been studied extensively and is reviewed in detail elsewhere (24). A 2013 genome-wide association study compared 188 patients with CHD with 188 healthy controls. Gene ontology enrichment analysis demonstrated that B cell activation, differentiation, and signaling genes were among the most prominently enriched in patients with CHD (25).

**Figure 1:**
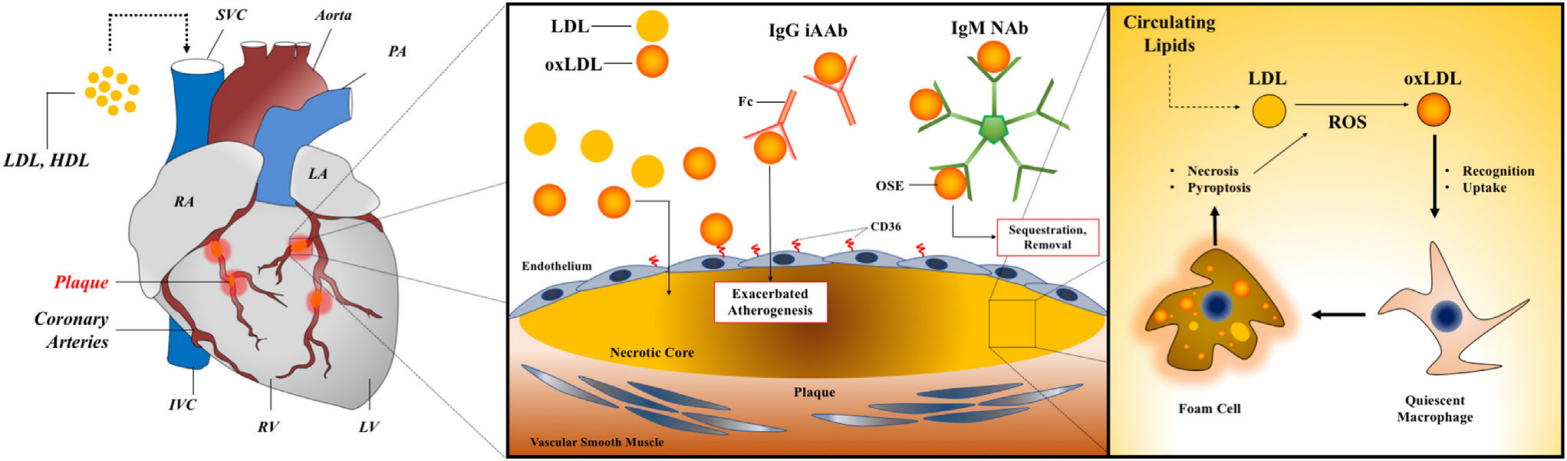
AAbs in myocarditis and dilated cardiomyopathy. Left panel: a diagram of cardiac anatomy with relevant structures labeled, including the coronary arteries and associated plaques. Middle panel: a generalized schematic for AAbs in atherogenesis showing opposing roles for B-1 cell-derived IgM NAb and B-2 cell-derived IgG iAAb. Right panel: foam cell formation and feed-forward inflammatory activation within vessel plaques through enhanced uptake of oxidized lipids during atherogenesis. Abbreviations: LDL, low-density lipoprotein; HDL, high-density lipoprotein; NAbs, natural antibodies; iAAb, induced autoantibodies; Ig, immunoglobulin; oxLDL, oxidized low-density lipoprotein; OSE, oxidation-specific epitope; RA, right atrium; LA, left atrium; IVC, inferior vena cava; SVC, superior vena cava; RV, right ventricle; LV, left ventricle; PA, pulmonary artery.

The predominant B cell subsets in mice and humans are B-1 and B-2 cells that produce NAbs and induced (adaptive) antibodies (iAb, iAAb when reactive with self-antigens), respectively. The dominant paradigm for understanding the role that B cells play during the natural history of ASCVD is based on opposing functions of B-1 and B-2 cells, with the former generally being disease-ameliorating and the latter disease-promoting. A schematic of how B-1 cell-derived NAbs and B-2 cell-derived iAAbs contribute to CAD is shown in Figure 1. The majority of studies that have contributed to construction of this paradigm have been derived from experimental atherosclerosis in mice, but clinical observations do indeed support this diametric model and are described in the following sections.

**Figure 2:**
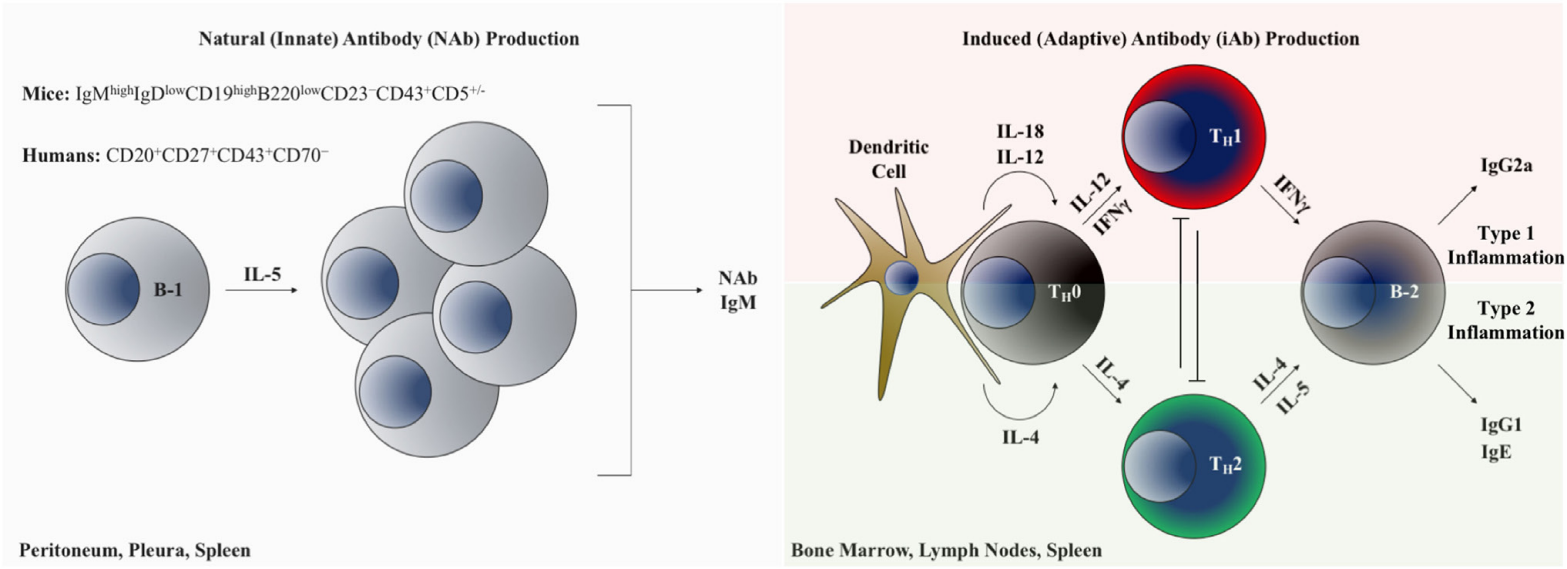
B-1 and B-2 cells as modulators of cardiovascular inflammation through AAb production. Left panel: B-1 B cells inhabiting the body cavities are interleukin-5 dependent and produce polyreactive natural antibodies, predominantly of the IgM isotype. Right panel: B-2 lymphocytes generate adaptive immunoglobulin under the control of inflammatory cytokine programming. Abbreviations: T_H_0, naive CD4^+^ T lymphocyte; T_H_1, type 1 inflammation-polarized CD4^+^ helper T lymphocyte; T_H_2, type 2 inflammation-polarized CD4^+^ helper T lymphocyte; IFN-γ, interferon gamma.

A comparison of B-1 and B-2 cells is shown in Figure 2. In mice, B-1 cells are identified and distinguished from the more common B-2 B cells based on lower expression of B220 and by the presence of CD43 (26). B-1 cells can be further subdivided into B-1a and B-1b subsets based on the presence or absence of CD5 expression, respectively (27). An analogous population of innate-like B-1 cells in humans that appears to have similar functional properties to those in mice is identified based on the following surface marker profile: CD20^+^CD27^+^CD43^+^CD70^−^ (28). Importantly, in humans, CD5 is promiscuously expressed on B-1 and B-2 cells in multiple contexts and is not a reliable distinguishing feature of these lineages (29). It is thought that in mice all three B cell subsets (B-1a, B-1b, and B-2) originate from distinct lineages (30), and are thus theoretically targetable through conditional and constitutive gene knockout studies to dissect their discrete functional differences. Clarifying the role of B cells in atherogenesis will require understanding the distinct versus overlapping functions of each subset in ASCVD. Table 1 provides a summary of the heterogeneous AAbs that are most actively studied in ASCVD with putative functional roles in the disease course highlighted.

**Table 1:** Summary of commonly studied autoantibodies in atherosclerotic cardiovascular disease.

Ab type	Antigen	Predominant isotype	Type of antigen	Promoting or restraining	References
Natural	Phosphatidylcholine	IgM	Oxidized phospholipid	Restraining	(64–69)
	Malondialdehyde-LDL	IgM >> IgG		Restraining	(48, 49)
	Phosphorylcholine (PC)	IgM (E06) and IgA (T15)		Restraining	(78–80)
	Cardiolipin	IgM and IgA >> IgG		Restraining	(52)

Induced	Endothelial cells (AECA^1^)	IgG	Unknown	Promoting	(138, 146, 147)
	Heat-shock protein-60/65	IgG	Protein metabolites	Promoting	(96–98)
	Apolipoprotein A-1	IgG		Promoting	(102, 103)

*^1^AECA, anti-endothelial cell antibody*.

## NAbs Restrain Atherosclerosis

Cardiovascular-reactive AAbs are produced in both homeostatic and disease states. NAbs are an important class of AAbs produced during homeostasis in the absence of cognate antigen encounter or infection. The biology of NAbs has been reviewed extensively elsewhere (31). In multiple experimental ASCVD models, NAbs have been shown to be disease-restraining (20, 32–35).

Natural antibodies are derived from B-1 cells (36) that are enriched in the spleen, bone marrow (BM), and body cavities (e.g., pleural and peritoneal) (31). In the absence of infection, the vast majority of serum IgM Ab in mice (between 80 and 90%) are NAbs (37). Characteristics of NAbs include (a) low-affinity for their target antigens relative to those resulting from the germinal center (GC) reaction and (b) polyreactivity with a wide array of structurally distinct cellular elements including autoantigens (e.g., elements of apoptotic cells and cell membrane components) and evolutionarily conserved microbial products (31). Of note, NAbs generally exhibit immunoglobulin-encoding gene segments in the germline configuration, indicating that their production is independent of somatic recombination and hypermutation (38, 39). Optimal development and production of NAb from B-1 cells requires IL-5 signaling (40, 41). The association between *Il5* gene hypomorphisms in humans with ASCVD provides a putative link to B-1 B cells and NAbs in this disease process (42). NAb reactivity with multiple self-derived antigens has been implicated in conferring this benefit, with many experimental studies focusing on NAb reactive to LDL derivatives (20, 32, 43–46). Despite multiple clinical studies supporting the ASCVD-restraining role for NAbs, studies demonstrating disease-promoting activity of NAbs have also been reported in both humans and in experimental ASCVD (47–50). Thus, it is not possible to reach generalizable conclusions regarding the role for NAb during the initiation and progression of ASCVD. With this caveat in mind, selected key findings that contribute to the understanding of NAb function in ASCVD are described below.

Plaque-accumulated lipid and cholesterol deposits are prone to oxidation, both spontaneously and enzymatically. Oxidation of plaque constituents renders them antigenic through formation of oxidation-specific (neo)epitopes (OSEs) when adducted to proteins within the plaques (51, 52). OSEs have been implicated in a variety of disease states, reviewed elsewhere (53). Some of the most widely studied endogenous OSEs within the context of ASCVD are derived from PL oxidation, including malondialdehyde (MDA) and phosphocholine (phosphorylcholine when functionally adducted) (53). These immunogenic OSEs subsequently induce an inflammatory reaction within the plaque and vessel wall vicinity. Employing a reductionist approach based on these observations, researchers have generated OSEs *in vitro*, such as copper-oxidized LDL (CuOxLDL), a model antigen containing heterogeneous OSEs generated through reacting purified LDL particles with copper sulfate (CuSO_4_) (54–56). CuOxLDL reagents have been useful for standardizing assays OSE-reactive AAb detection assays (56) and for clarifying OSE-induced immune responses through immunization in experimental models of ASCVD (57).

Phosphatidylcholine is ubiquitous in mice and humans and is a component of cell membranes and cholesterol particles (both HDL and LDL). It readily undergoes enzymatic oxidation by platelet-activating factor-acetylhydrolase (LP-PLA2) to yield immunogenic phosphorylcholine (PC) (58), an OSE that has been demonstrated to be an important inflammatory mediator in the setting of ASCVD, functioning through activation of T cells, monocytes, and endothelial cells following protein adduction (51, 59–63). In humans, serum levels of PC-reactive natural IgM (anti-PC-IgM) were inversely correlated with the risk of atherosclerosis, vein-graft stenosis, and stroke in the general population and also in patients with systemic lupus erythematosus (64–69). Additional studies in humans have demonstrated associations between alternative OSEs and their role in atherogenesis. For example, MDA, an OSE produced from the breakdown of polyunsaturated fatty acids, is a highly reactive moiety that readily forms immunogenic protein adducts and is recognized by circulating IgM AAbs. Multiple observational studies in humans have demonstrated inverse correlations between MDA-reactive IgM AAbs and atherosclerotic disease. Specifically, low levels of MDA-reactive NAbs are correlated with increased carotid intima-to-media thickness (IMT, a clinical measure of atherosclerosis determined using angiography) and increased risk of coronary artery stenosis (35, 48). These studies provide preliminary evidence that strategies for enhancing NAb production may be beneficial for combatting ASCVD.

Studies in *Apoe*^−/−^ and *Ldlr*^−/−^ mice have provided evidence for the disease-restraining role for NAbs during atherosclerosis initiation and progression and provide researchers with the ability to dissect the cellular and molecular pathways mediating their production. Splenectomized *Apoe*^−/−^ mice developed more aggressive lesions than intact *Apoe*^−/−^ control mice, a phenotype that could be rescued through adoptive transfer of B-1a cells (34). Importantly, this protection depended on the ability of the transferred B-1 cells to secrete IgM. Recently, B-1b cells were shown to be sufficient for atheroprotection in *Apoe*^−/−^ mice *via* OSE-specific NAb production (70). The authors provided evidence implicating DNA-binding protein inhibitor 3 (Id3) as a negative regulator of B-1b cell development–conditional deletion of *Id3* in B cells using *Cd19-Cre* on the *Apoe*^−/−^ background led to increased B-1b cell numbers, increased titers of oxLDL-NAb, and decreased atherosclerotic lesion formation. The study was buttressed by the authors’ identification of a hypomorphic *Id3* polymorphism in humans that leads to elevated B-1 cell numbers and oxLDL-NAb levels. Interestingly, the same group previously reported that *constitutive* deficiency of *Id3* significantly exacerbated atherogenesis (71), thus implicating potential alternative functions for *Id3* in non-B cell populations during the natural history of ASCVD.

Using spleens from *Apoe*^−/−^ mice, researchers cloned of 13 oxLDL-reactive NAb (designated “E0” Ab) (44). The E06 antibody generated from these studies recognizes phosphorylcholine-adducted oxidized phospholipids (oxPL) and not free PC or reduced/native PLs (72). *In vitro* studies using the E06 antibody demonstrated its ability to prevent macrophage uptake of oxLDL, an important element of foam cell formation during atherogenesis (73, 74). Clone E06 was later shown to competitively inhibit CuOxLDL binding to CD36 [a member of the scavenger receptor (SR) family of proteins that mediates oxLDL uptake], demonstrating not only that CD36 is a receptor for oxPL but also that oxPL-specific NAbs inhibit CD36-mediated oxLDL uptake (75), which may then interfere with CD36-mediated foam cell formation (74). The more recent observation that CD36 ligands promote inflammatory responses through activation of a TLR4/6 signaling cascade provides further insight into potential pathways by which NAb (E06 in particular) may mediate ASCVD-protective effects (76).

The B-1 cell-derived T15 IgA NAb clone has been studied extensively and was previously shown to confer enhanced *Streptococcus pneumoniae* immunity in mice through recognition of PC in the *Streptococcus* capsule (77, 78). Intriguingly, the antigen-binding domains of E06 and T15 are identical and differ only in isotype (79). Immunizing *Ldlr*^−/−^ mice with preparations of *S. pneumoniae* significantly elevated NAb IgM titers and reduced plaque development, thus demonstrating the presence of molecular mimicry between *S. pneumoniae* and oxLDL in addition to a potential mechanism by which NAbs generally restrain ASCVD progression. In addition, this and other studies have provided evidence for a potential vaccine for atherosclerosis prevention based on enhancing NAb production (80). Later studies showed that passive immunization of *Apoe*^−/−^ mice with monoclonal T15 antibody resulted in significant reductions in the development of vein-graft atherosclerosis without altering serum cholesterol levels (81), establishing the potential efficacy of NAb-based therapies as a treatment for ASCVD.

Many of the molecular and cellular mechanisms that underlie the association between NAb and atheroprotection still need more investigation. A number of putative, and non-mutually exclusive, mechanisms have been proposed based on experimental observations. These include binding and recognition of oxidized lipid adducts on the surface of apoptotic cells, thereby promoting plaque macrophage- and/or dendritic cell-mediated recognition of dead/dying cell debris (43), potentially in a process dependent on the complement component, C1q (82). Others have demonstrated a role for oxLDL-reactive NAb-mediated inhibition of endothelial cell (EC) activation and IL-8 (CXCL8) secretion in response to stimulation by dying cells (83). EC expression of CD36 has emerged as a potential target in vascular disease due to its ability to mediate pro-inflammatory activation, and immune cell recruitment (84, 85). It is likely that endothelial CD36 is involved in the ameliorative effects of NAbs in atherogenesis. One report indicates that oxLDL-NAbs restrain atherogenesis in the absence of altered plaque apoptotic cell clearance (34). Thus, it is highly likely that NAbs have additional functions in ASCVD that remain to be defined.

## iAAbs may Promote Atherosclerosis

The most prevalent antibody isotype in human serum is adaptive/induced IgG produced by B-2 cells. Within the context of CVD, iAAbs of the IgG isotype have been studied extensively (24). Like atherosclerosis-associated NAb produced during homeostatic conditions, iAAbs produced in the setting of inflammation and/or infection also play a role in atherogenesis. The specific functions of iAAbs are much less clear, due partly to contradictory conclusions within the literature. Unlike homeostatically produced NAb, production of iAAbs requires the concerted interaction of multiple cell types (namely B- and T-lymphocytes and antigen presenting cells) and inflammatory signals (cytokines) to transform a naive B cell into a class-switched, somatically hyper-mutated, and antibody-secreting plasma cell.

By contrast to the relative breadth of data demonstrating an atheroprotective role for B-1 cell-derived NAb, the role for B-2 cell-derived iAAb in atherosclerotic disease remains unresolved. Early studies of B cells in ASCVD initially implicated a disease-restraining role for B-2 cells: splenectomized *Apoe*^−/−^ mice displayed an exaggerated atherosclerotic phenotype that could be rescued through adoptive transfer of splenocytes (86). In agreement with these observations, it was also shown that atherogenesis was amplified in *Ldlr*^−/−^ mice reconstituted with BM from B cell-deficient animals (*Ighm*^−/−^, encoding μMT), relative to those reconstituted with B cell-replete, wild-type BM (87). In contradiction to the disease-restraining role for B-2 cells suggested by these studies, it was later shown that systemic B-2 cell depletion with anti-CD20 mAb in either *Apoe*^−/−^ or *Ldlr*^−/−^ mice significantly reduced atherosclerotic lesion formation (88, 89). While multiple explanations could explain these disparate observations, including differences in housing conditions and microbiota (90), contradictory conclusions exist and are in need of further study. It is important to acknowledge the phenotypic and functional diversity of B-2 cells, which include marginal zone, follicular, and regulatory B cell (B_regs_) subsets (91). Clarification of the disparate observations highlighted above will likely involve determining the discrete roles each of these B-2 cell subsets plays during the natural history of ASCVD.

Similar to B-1 cell-derived NAb, iAAbs reactive to OSE have been reported in both human atherosclerosis and animal models of it (92–95). Demonstration of a clear correlation between serum OSE-reactive IgG and disease severity in humans has been challenging; while some studies have shown weak positive correlations, and others have shown none. In experimental murine atherosclerosis, OSE-reactive IgG titers correlate with plaque burden, increasing during plaque growth and decreasing during plaque regression (94). This correlative observation says little about the specific role for OSE-reactive IgG AAbs throughout the disease course, however. While many experimental observations have demonstrated a pro-atherogenic role for OSE-reactive IgG AAbs, their functional roles in ASCVD are far from resolved.

In addition, strategies investigating vaccination for generating adaptive antibody responses to ASCVD-associated epitopes have also shown observations that conflict with the putative disease-promoting role for iAAbs in ASCVD (49, 57). Repeated immunization of *Ldlr*^−/−^ mice with MDA-adducted LDL (MDA-LDL) or native LDL over a period of 7 weeks followed by atherogenesis induction using a high-fat diet demonstrated a significant reduction in atherosclerotic lesion formation in both cases (49). While less dramatic in the setting of native LDL immunization, both immunization strategies significantly reduced lesion formation relative to saline-injected control animals. Significantly elevated titers of isotype-switched Ab with specificity to oxidized lipid products were only observed in the setting of MDA-LDL immunization. Both type 1 inflammation-polarized CD4+ helper T lymphocyte- and type 2 inflammation-polarized CD4+ helper T lymphocyte-associated antibody titers (IgG2a and IgG1, respectively) were increased in this setting, and were inversely correlated lesion development. Thus, these observations complicate assigning an ASCVD-promoting role for iAAbs.

In addition to OSEs, HSP-60 is an autoantigen that has been of interest to the atherosclerosis field. In the setting of atherosclerotic inflammation, endothelial cells upregulate expression of HSP-60 which displays structural similarity (i.e., molecular mimicry) with HSP-65 from *Mycobacterium* and *Chlamydia spp*. (13). In the context of before exposure or infection, the existence of an anti-HSP-65 antibody response provides a mechanism for induction of autoimmunity in the setting of atherosclerotic inflammation leading to upregulation of endothelial HSP-60. In support of a pro-inflammatory role for HSP-65 AAbs in atherogenesis, induction of arterial inflammation in normocholesterolemic rabbits was observed following immunization with HSP-65 without alterations in serum cholesterol (96). In addition, *Ldlr*^−/−^ mice on regular chow developed atherosclerotic lesions following intraperitoneal injections of anti-HSP-65-IgG (97). While one epidemiological study in humans demonstrated HSP-65-reactive IgG titers correlated with atherosclerosis severity (as measured by carotid IMT) (98), and another demonstrated no correlation (99). Further exploration is needed to clarify these disparate observations.

Antibodies against apolipoprotein A-1 (ApoA1), a main protein constituent of HDL, were initially observed in patients with systemic inflammatory diseases such as right atrium (RA) (100). While HDL levels are commonly thought of as being atheroprotective, induced IgG antibody responses to immunogenic products of ApoA1 degradation have demonstrated positive correlations with atherogenesis. HDL promotes lipid clearance and disposal through reverse cholesterol transport. Whether anti-ApoA1 Ab interfere with this process has not been determined. It was shown in patients with RA that circulating ApoA1-reactive IgG antibody titers are superior predictors of major cardiac events relative to more than 15 biomarkers tested in the study including serum HDL, LDL, triglycerides, and CRP (101). Using patient studies, it has been hypothesized that anti-ApoA1 IgG promotes inflammatory activation through stimulation of a toll-like receptor 2-TLR4-NFκB signaling axis in innate immune cell populations (102). Later, it was observed in humans that resting heart rate (a prognostic marker used in assessing patients following MI) was inversely correlated with anti-ApoA1 IgG titers. Expanding on these observations, when rat cardiomyocytes were cultured in the presence of aldosterone with or without anti-ApoA1 IgG Ab, and spontaneous contraction was shown to decrease in an anti-ApoA1 IgG dose-dependent fashion (103). While additional studies are necessary to dissect the mechanisms that underlie these observations, anti-ApoA1 IgG AAbs also appear to be a potential therapeutic target in ASCVD treatment.

A hallmark of late-stage atherosclerotic disease in humans and experimental atherosclerosis is the development of arterial tertiary lymphoid organs (ATLOs) in the adventitia at the sites of plaque formation (104–106). An attractive hypothesis to explain their genesis rests on a compensatory response to chronic inflammatory stimulation. The definitive functional role of ATLOs in the context of atherosclerotic disease has long eluded the CV research community. Recently, multiple studies have attempted to address this. Using *Apoe*^−/−^ mice on a Western diet, the authors elegantly demonstrate that ATLO contain a T-follicular-helper (T_FH_)-GC B cell-axis that that governs lesion formation and promotes exacerbated disease. The authors additionally demonstrated the ATLO T_FH_-GC B cell-axis is restrained through CD8^+^ regulatory T cells that are restricted to the non-classical major histocompatibility complex Qa-1 [the mouse ortholog of human leukocyte antigen-E] (105). Another study from the same year reached the opposite conclusion about the function of ATLO in *Apoe*^−/−^ mice (106). Therein, the authors concluded that ATLO formation restrains atherosclerosis; disruption of ATLO formation through conditional deletion of the lymphotoxin-β receptor in vSMCs exacerbated lesion formation (106). The humoral consequences of disrupting ATLO formation were beyond the scope of the study in question. Nonetheless, the opposing conclusions reached by these two studies are in need of further clarification. Resolving the role of ATLO in ASCVD will contribute to the understanding of humoral immunity and AAbs in this context.

## Myocarditis and DCM: The Role of AAbs

Myocarditis is inflammation of the myocardium and its most common sequela is DCM (107). DCM is the most common cause of heart failure in children and young adults, and it is thought that as many as one in three cases of myocarditis progress to DCM (108). While not all cases of myocarditis result in DCM and while not all cases of DCM are the result of myocarditis, there exists a clear link between the two disease manifestations in a significant proportion of cases. Due to this connection, substantial research emphasis has been placed on understanding DCM immunopathogenesis and how it progresses from myocarditis. It is currently believed that autoimmune-mediated DCM represents the major subtype of the disease (109) with emerging evidence that type 3 inflammatory signals [i.e., those mediating CD4^+^ T-helper (T_H_) polarization toward a T_H_17 phenotype] play a critical role in its pathogenesis (110–114).

Clinical studies have associated multiple AAbs with myocarditis and DCM. This topic has been reviewed in extensive detail elsewhere (115). IgG AAbs directed against the β1-adrenergic receptor (β1AR) were detected in the sera of DCM patients and shown to inhibit catecholamine binding when cultured with rat cardiomyocytes *in vitro*, whereas sera from patients with ischemic CM, VHD, and healthy controls demonstrated no effect (116). A later study using a synthetic peptide derived from an extracellular domain of β1AR demonstrated elevated anti-β1AR AAbs in the sera of DCM patients relative to controls (31 versus 12%, respectively) (117). Interestingly, anti-β1AR AAbs were also detectable in the healthy control group, demonstrating that the mere presence of anti-β1AR is not predictive of pathology. When Japanese white rabbits were immunized with a synthetic peptide corresponding to an extracellular domain of the β1AR, induction of anti-β1AR IgG production was observed (118). Purified anti-β1AR IgG from these animals inhibited catecholamine responsiveness when cultured on rabbit cardiomyocytes. At 6-months post-immunization, cardiac hypertrophy and abnormal hemodynamics were seen. Additional analyses indicated evidence of anti-β1AR AAb-mediated adrenergic over-stimulation leading to compensatory downregulation of β1AR expression, and concomitant upregulation of proteins that inhibit β-adrenergic signaling, thus laying the groundwork for a disease-exacerbating positive feedback loop. An additional study in a limited patient population demonstrated that a significant proportion (36%) of DCM patients with circulating anti-β1AR IgG also exhibited elevated anti-M2-muscarinic receptor (M2AChR) IgG AAbs (119). Relative to serum samples from control patients, anti-M2AChR IgG AAbs were significantly elevated in the context of DCM (39% in DCM versus 8% in controls) (119). In the myocardium, muscarinic and adrenergic signaling exert opposing effects, with muscarinic signaling exhibiting negative inotropic and chronotropic effects and adrenergic signaling doing the opposite. Overstimulation and desensitization of each pathway have been postulated as a functional consequence of circulating anti-M2AChR and anti-β1AR AAbs, ultimately leading to heart failure (118, 120). Additional studies confirming these hypotheses are needed, however. Additional cardiac-related autoantigens with established AAb reactivity in human DCM include cTnI (11, 121–123), mitochondrial M7 (124), adenine-nucleotide transporter (ANT) (125), Na-K ATPase (126), actin (127), acetylcholine receptor (128), α/β Myosin heavy chain (129, 130), myosin light chain-1 (127), HSP-60 (131), sarcoplasmic reticulum Ca^2+^-ATPase (SR-Ca^2+^-ATPase) (132), laminin (133), and tropomyosin (127). A summary of the most studied antigens observed in myocarditis and cardiomyopathy can be found in Table 2. The primary mechanism by which AAbs exacerbate disease in DCM remains unknown, however, and alternative explanations exist to explain their roles that include to target neutralization and adaptation to persistent stimulation.

**Table 2:** Summary of the most commonly studied cardiac-related autoantibodies in myocarditis and cardiomyopathy.

Antigen	Proposed pathological mechanism	References
Acetylcholine receptor	Negative inotropy and bradycardia	(128)
Actin	Undefined	(127)
Adenine-nucleotide transporter	Metabolism inhibition	(125)
β1-adrenergic-R	Negative inotropy	(116, 117)
Heat-shock protein-60	Increased recognition clearance of stressed cardiomyocytes	(127, 131)
Laminin	Undefined	(133)
M2 muscarinic AChR	Negative inotropy	(119, 120)
Mitochondrial M7	Undefined	(124)
α/β Myosin heavy chain	Negative inotropy and failure of thymic self-tolerance	(129, 130)
Myosin light chain-1	Undefined	(127)
Na-K ATPase	Arrhythmogenicity	(126)
Sarcoplasmic reticulum-Ca-ATPase	Metabolism alterations	(132)
Tropomyosin	Undefined	(127)
Troponin	Negative inotropy	(11, 121–123)

Again, underscoring the inflammatory nature of this DCM, as many as one out of three cases of myocarditis ultimately progresses to DCM. The concept of molecular mimicry is central to the understanding of autoimmune responses to cardiac antigens and multiple infectious agents have been identified with elements bearing epitope similarity to them (134). Known infectious causes of human myocarditis that exhibit molecular mimicry of cardiac antigens include *Trypanosoma cruzi* (135), parvovirus B19 (136), coxsackievirus (15), and *Borrelia* spp. (137). In each case, cardiac myosin appears to contain dominant epitopes bearing structural similarity to pathogen-derived antigens.

Rheumatic heart disease (RHD) provides a prototypical example of molecular mimicry in CM. In RHD, untreated and repeated infections with *Streptococcus pyogenes* [group A strep (GAS)] may lead to acute rheumatic fever characterized by a constellation of symptoms resembling many rheumatic conditions including polyarthritis, in addition to carditis (138). RF progresses to chronic RHD in as many as 50% of patients (139). Cross-reactivity between GAS and components of cardiac proteins is currently accepted as a key driver of RHD (140). Determination that M proteins (one of the major virulence factors expressed by GAS) exhibit structural similarity with cardiac myosin provided critical insight into the nature of RHD (141). Since, it has been shown that components of GAS [including its carbohydrate antigen and *N*-acetyl-β-d-glucosamine (GlcNAc) (142, 143)] display molecular mimicry with additional cardiac antigens, such as laminin (144), tropomyosin (145), the endothelium (146–148), and others, including those restricted to the cardiac valves (144). Generation of adaptive Ab responses to infections with cross-reactivity to cardiac antigens is a critical element of post-infectious myocarditis and its common sequela DCM.

Much of the understanding of infectious myocarditis and DCM has been garnered from animal models of experimental autoimmune myocarditis (EAM). Inflammatory HD with many histologic features of RHD (including pan-carditis, granulomatous lesion formation, the presence of Anitschkow cells, and late-stage valvular scarring) was accomplished by immunizing mice *via* intraperitoneal injections of a sonicated preparation of GAS (149). Refining the experimental approach, the Cunningham group developed a rat model of RF/RHD based on immunization of Lewis rats with purified M protein (150–152). In addition to myocardial inflammation, cardiac valve pathology was also observed in these studies. In addition, identification of CD4^+^ T cells with M protein cross-reactivity lent further insight into mechanisms by which infection may induce an adaptive AAb response *via* the support of CD4^+^ T cell help. The specific cellular and molecular mechanisms by which cardiac-reactive AAbs mediate tissue destruction in these model systems (and in human RHD) remain unclear, however. Future investigation of EAM models that utilize conditional and constitutive gene deletion will be useful for mechanistic studies and clarification of these observations.

Multiple viruses (enteroviruses, most commonly) have been established as causative agents of myocarditis/DCM. The most well-studied of these is coxsackievirus B3 (CVB3), a cytolytic enterovirus with cardiotropism (153). The presence of detectable enteroviral genomic material and enteroviral-reactive Ab has been observed in as many as 70% of DCM patients (15). In CVB-induced EAM, it has long been known that anti-cardiac myosin AAbs are generated during the disease course and that cardiac myosin-reactive AAb titers correlate with myocarditis severity (154). It is unclear what functional role anti-cardiac myosin AAbs play during the course of viral myocarditis/DCM; a lack of cross-reactivity between cardiac myosin-reactive AAbs and CVB3 was reported, thus contradicting the mimicry hypothesis as a driver of CVB3-myocarditis/DCM (155). It has been postulated that, rather than participating as active promoters of enteroviral-induced cardiac damage, AAbs generated during CVB3 infection are bystanders in the disease process, with their titers reflecting the degree of tissue damage (153). CVB3 has also been shown to share moderate sequence homology to mitochondrial ANT, a postulated alternative target of molecular mimicry during CVB3-induced EAM (156). In a limited cohort, anti-ANT AAbs were observed in 94% of DCM patients (125). Additional work is needed to determine whether this represents a clinically relevant antigen for AAb targeting in viral myocarditis/DCM.

Spontaneous endocarditis and valvular carditis occur with complete penetrance in the T cell receptor (TCR) transgenic K/B.g7 mouse line (also referred to as K/BxN in some studies), without immunization or infection (157). Thus, this model provides a useful tool for dissecting the cellular and molecular mechanisms that underpin cardiac pathology in the setting of sterile systemic inflammation. K/B.g7 mice exhibit expression of a transgenic TCR termed “KRN” that recognizes a peptide derived from the self-protein glucose-6-phosphate-isomerase (GPI, a ubiquitous metabolic enzyme) presented in the context of the I-A^g7^ major histocompatibility complex II molecule from the non-obese-diabetic mouse strain (158–160). Systemic GPI-specific T cell activation leads to production of high-titer anti-GPI IgG AAbs. K/B.g7 mice develop erosive polyarthritis, endocarditis, and fibrotic valvular carditis with a left-sided predilection, primarily affecting the mitral valve (MV) (157, 161). It was demonstrated that macrophages are the key cellular mediators of valve pathology in K/B.g7 mice; animals treated systemically with macrophage-depleting clodronate liposomes were protected from MV disease (MVD) (161). Using constitutive gene deletion approaches, our group demonstrated that activating IgG receptors (FcγRs), specifically FcγRIII (CD16) and FcγRIV (CD16.2), act redundantly and are required for MVD; protection from disease occurs only in the absence of both (161). These results support a model whereby circulating IgG AAbs mediate cardiac inflammation through macrophage activation downstream of activating FcγR-mediated recognition of circulating IgG AAbs. Additional studies employing conditional gene deletions to dissect the key functional consequences induced in macrophages following IgG AAb recognition have provided important insight into mechanisms by which AAbs contribute to experimental VHD (162). Samples from patients with RHD were used to demonstrate correlations of the authors’ experimental observations to human inflammatory VHD.

## Future Directions

Mechanistic insight is needed to better understand the role for AAbs in CV pathology. Studies pursuing mechanistic rather than descriptive and/or correlative insight will be critical for rectification of the seemingly conflicting observations, and conclusions that have been seen and reached. Identification of therapeutically targetable elements of AAb generation in CVD will require cellular and molecular mechanistic insight. Gene deletion approaches, both constitutive and conditional, for experimental dissection of the pathways by which AAbs function in the heterogeneous manifestations of CVD will likely prove useful to this field. Significant questions that remain unanswered include: what determines the quality (i.e., isotype and affinity) and quantity (i.e., circulating titers) of an Ab response in the setting of CV inflammation, and how do these antibody responses engage additional immune cells to restrain or promote CV immunopathology. Finally, how does the interplay between CVD-associated Ab and the discrete inflammatory lineages with which they engage ultimately interface with the underlying tissue parenchyma (e.g., myocardium and blood vessel wall) to promote or restrain detrimental CV remodeling (e.g., plaque formation and fibrosis). Employing lineage-specific gene deletion approaches (e.g., Cre-loxP recombination) will undoubtedly prove illuminating. Finally, progress within this field will be well served by demonstrating clinical relevance through correlation of experimental observations to human disease. In addition to their rarity, samples from human disease states are often logistically complicated to acquire. Despite this, the potential insight that can be gained from correlations between experimental disease models and human pathology cannot be underscored. Future progress in this field will ultimately be made through clinical translation. Thus, complementing mechanistic work in experimental models with observations in inflammatory human CV pathology will be critical.

## Conclusion

There is substantial evidence for the disease-modulating role that AAbs have in CVD. Despite the breadth of evidence, there is little consensus regarding the specific functions that AAbs have in the various forms of CVD in which they are implicated, and numerous conflicting observations and hypotheses have been reported. A large majority of studies have been observational or correlative, rather than mechanistic, and therefore have not translated into therapeutic strategies for human CVD. The field now needs to focus on how these AAbs engage particular molecular and cellular immune components to influence disease severity; the insights provided by this approach will point the way to new therapeutic options.

## Author Contributions

LM and BB determined the scope of this review, wrote the manuscript, and revised the manuscript. LM generated the figures and tables.

## Conflict of Interest Statement

The authors affirm that this manuscript was generated in the absence of any material or financial conflict of interest that may have potentially influenced its content.
